# APOC3 as a potential prognostic factor for hepatitis B virus-related acute-on-chronic liver failure

**DOI:** 10.1097/MD.0000000000041503

**Published:** 2025-02-07

**Authors:** Bo Wang, Li Qiang, Geng Zhang, Wen Chen, Yunjian Sheng, Gang Wu, Cunliang Deng, Shan Zeng, Qian Zhang

**Affiliations:** a Department of Infectious Diseases, The Affiliated Hospital of Southwest Medical University, Luzhou, Sichuan, China; b Department of Respiratory and Critical Care Medicine, The Affiliated Hospital of Southwest Medical University, Luzhou, Sichuan, China.

**Keywords:** ACLF, APOC3, DIA, HBV, prognosis

## Abstract

Acute-on-chronic liver failure (ACLF) is the major cause of mortality in patients infected with the hepatitis B virus (HBV); however, early determination of the prognosis of patients with HBV-ACLF is insensitive or limited. This study aimed to analyze differentially expressed proteins in the plasma of patients with HBV-ACLF using data-independent acquisition mass spectrometry to provide a reference for short-term prognosis. Fifty HBV-ACLF patients and 15 healthy controls were enrolled in this study. Of these, 10 patients with HBV-ACLF and 5 healthy volunteers participated in data-independent acquisition-based proteomics and the potential core proteins were screened out via bioinformatics. Apolipoprotein C3 (APOC3) was selected and quantified by enzyme linked immunosorbent assays in all patients. And the area under the curve (AUC) was calculated to evaluate the value of APOC3 in the diagnosis and prognosis of patients with HBV-ACLF. A total of 247 differentially expressed proteins were identified in the serum of patients in the HBV-ACLF and normal control groups. A total of 148 proteins were upregulated and 99 proteins were downregulated in the HBV-ACLF group compared with those in the normal group. The expression level of APOC3 was 1.65 ± 0.44 mg/mL in patients with HBV-ACLF, which was obviously lower than the normal controls (2.04 ± 0.22 mg/mL) (*P* < .001) (AUC was 0.766, with a sensitivity of 62%, and specificity of 93.3%). The expression level of APOC3 was 1.38 ± 0.44 mg/mL in the non-survival group, which was obviously lower than the survival group (1.83 ± 0.35 mg/mL) (*P* < .0001) (AUC was 0.780, with a sensitivity of 50%, and specificity of 96.7%). APOC3 is associated with short-term prognosis of patients with HBV-ACLF and can be used as a potential prognostic biomarker in patients with HBV-ACLF.

## 1. Introduction

Acute-on-chronic liver failure (ACLF) is a clinical syndrome characterized by rapid deterioration of liver function in association with liver and extrahepatic failure in patients with chronic liver disease.^[1,2]^ One of the main causes of ACLF is chronic hepatitis B virus (HBV) infection and more than 70% in ACLF cases.^[3,4]^ ACLF has a rapid onset and rapid progression, which can cause multiple organs in a short period of time, and its short-term mortality rate is as high as 50% to 90%.^[5–7]^ Currently, the prognosis of HBV-ACLF is poor, and ideal treatment is lacking. The main clinical treatments for HBV-ACLF include antiviral nucleoside analogs, artificial liver support devices, therapeutic plasma exchange, and liver transplantation.^[8–11]^ Integrative approaches to internal medicine can only alleviate the disease, block progression, and improve the prognosis in the early stages of HBV-ACLF. Liver transplantation is considered the only curative therapy for patients with HBV-ACLF^[4]^ and is limited by organ shortage, high cost, and post-transplantation immune rejection, which contribute to a lower survival rate. Early and accurate assessment of the condition of patients with HBV-ACLF can help clinicians determine the best treatment plan, guide donor liver allocation, and improve patient outcome.

Several prognostic predictors and predictive scoring models can be used to assess disease severity and prognosis in patients with HBV-ACLF. These classic prognostic models include the model for end-stage liver disease (MELD) score, MELD-sodium (MELD-Na) score, Sequential Organ Failure Assessment (SOFA), Acute Physiology and Chronic Health Evaluation, chronic liver failure-Sequential Organ Failure Assessment (CLIF-SOFA) score, Chronic Liver Failure Consortium ACLF (CLIF-C-ACLF) score, and the Chinese Group on the Study of Severe Hepatitis B ACLF score.^[12–15]^ However, these predictive scoring models are too complex to assess disease severity and guide therapy. Therefore, it is important to identify new simple and reliable markers to assess the disease severity and prognosis in patients with HBV-ACLF.

Proteomics technology, which is a comprehensive and high-throughput analytical approach, has revolutionized biomedical research and brought tremendous changes to areas such as disease diagnosis, drug discovery, and personalized medicine. Proteomics is a powerful tool that enables the identification and quantification of novel biomarkers for various diseases.^[16]^ Proteomic approaches focus on identifying new biomarkers for the diagnosis, prognosis, and targeted treatment of various diseases. Data-independent acquisition mass spectrometry (DIA-MS) is emerging as a technology that combines deep proteomic coverage capabilities with quantitative consistency and accuracy.^[17]^ DIA-MS has demonstrated high reproducibility and precision in protein quantification, and has been applied to the study of diseases.^[18]^ However, proteomic methods for the discovery of HBV-ACLF biomarkers in the serum are limited. In our previous study and in this study, we used DIA-MS proteomics and bioinformatics analysis to identify and quantify serum proteins that were differentially expressed in patients with HBV-ACLF compared with healthy individuals. Furthermore, we validated the candidate biomarker, apolipoprotein C3 (APOC3), which has potential diagnostic or prognostic value in patients with HBV-ACLF.

## 2. Materials and methods

### 2.1. Patient recruitment and sample collection

All clinical data collected from the clinical records of patients in the Department of Infectious Disease of the Affiliated Hospital of Southwest Medical University between January 2021 and April 2022 were used in this study. The Chinese diagnostic criteria for HBV-ACLF were^[1]^: (1) chronic liver disease and hepatitis B virus infection for > 6 months. (2) Serum total bilirubin (TB) ≥ 12 mg/dL and international normalized ratio ≥ 1.5. The exclusion criteria were as follows: (1) combined with other hepatitis virus infections (including hepatitis A, C, D, E, cytomegalovirus, Epstein–Barr virus, and other hepatotropics. viruses). (2) Combined autoimmune, alcoholic, drug-induced, and fatty liver diseases. (3) Malignant tumors. (4) Other serious diseases (heart failure and hyperthyroidism). (5) Pregnancy or lactation. (6) Hematological disease. (7) Septicemia. Sixty-five clinical samples were included in this study. Fifty patients with HBV-ACLF (HBV-ACLF group, n = 50) and 15 healthy volunteers (control group, n = 15) were included in the study. All patients with HBV-ACLF in the study were followed for 90 days or until death. The blood samples were collected, centrifuged, and separated. Plasma samples were frozen at −80 °C. This study was approved by the Ethics Committee of the Affiliated Hospital of the Southwest Medical University (approval number: KY2021014). All the participants signed an informed consent form.

### 2.2. DIA quantitative proteomic analysis

Ten plasma samples from the HBV-ALCF group and 5 plasma samples from the control group were randomly selected for proteomic analysis by DIA-MS. We used the tandem mass spectrometer Q-Exactive HFX (Thermo Fisher Scientific, San Jose, CA) for the data-dependent acquisition detection mode (DDA mode) and data-independent acquisition detection mode (DIA mode). The main parameters of the DDA mode were set as follows: (1) MS: 350 to 1500 m/z; 120,000 resolution; maximal injection time (MIT) 50 ms; NCE 28; (2) HCD-MS/MS: 30,000 resolution; MIT 100 ms; dynamic exclusion duration 30 s. The main parameters of DIA mode were set as follows: (1) MS: 400 to 1250 m/z; 120,000 resolution; MIT 50 ms; (2) HCD-MS/MS: 30,000 resolution; automatic MIT; dynamic exclusion duration 30 s; stepped NCE: 22.5, 25, and 27.5.

### 2.3. Data analysis

MaxQuant (http://www.maxquant.org)^[19]^ was used to identify the DDA data and subsequent DIA analysis. A false discovery rate of ≤ 1% was used to build the final spectral library. MSstats^[20]^ were used for the statistical evaluation of significant differences in proteins. Differential protein screening was performed according to the threshold of a fold-change ≥ 2 and *P*-value < .05, as the criteria for determining significant differences.

### 2.4. Bioinformatics analysis

To further understand the functions of differentially expressed proteins (DEPs) in ACLF. Functional classification of these DEPs was performed using Gene Ontology (GO) analysis (http://www.geneontology.org), and protein–protein interaction (PPI) network analysis was performed using the STRING database 11.0 (http://string-db.org/).

### 2.5. ELISA validation

Changes in the expression of the selected proteins identified by DIA analysis were confirmed. In this study, APOC3 was selected and quantified by ELISA in all patients consisting of fifty HBV-ACLF patients and 15 healthy controls. Samples and reagents were prepared according to the manufacturer’s instructions for the APOC3 ELISA Kits. ROC curves were drawn using GraphPad Prism (version 8.0) and the area under the curve was calculated to evaluate the value of APOC3 in the diagnosis and prognosis of patients with HBV-ACLF.

### 2.6. Statistical analysis

The data obtained from the patients’ clinical information and ELISA were analyzed using IBM SPSS Statistics 26.0, and graphs were drawn using GraphPad Prism 8.0. Continuous data are expressed as mean ± standard deviation (SD), and data with a skewed distribution are expressed as medians (P25, P75). The independent *t* test and nonparametric Mann–Whitney *U* test were used for statistical analyses. Pearson chi-square test or Fisher exact test was used to compare categorical data. Statistical significance was set at *P* < .05.

## 3. Results

### 3.1. Clinical characteristics and demographics

In total, 50 HBV-ACLF patients and15 healthy controls were enrolled in this study. Of these, 10 patients with HBV-ACLF and 5 healthy volunteers participated in DIA-based proteomics. The baseline characteristics of the study participants are shown in Table 1. The neutrophil cell count, alanine transaminase, aspartate transaminase, and TB levels were significantly higher in the HBV-ACLF group than in the normal control group. Hemoglobin and platelet levels were significantly lower in the HBV-ACLF group than in the normal group. There was no statistically significant difference in creatinine concentration and white blood cell count between the 2 groups (Table 1).

**Table 1:** Baseline characteristics of patients with HBV-ACLF group and normal group (NC).

Features/groups	HBV-ACLF (n = 50)	NC (n = 15)	*P*-value
Sex (Male/Female)	39/11	10/5	.763*
Age (years)	50.78 ± 12.44	36.87 ± 4.83	<.001
WBC (10^9^/L)	6.88 (5.82–8.79)	6.61 (4.76–7.33)	.188
NEU (10^9^/L)	4.61 (3.71–6.52)	3.54 (3.04–4.55)	.009
HGB (g/L)	128.30 ± 21.73	146.07 ± 15.74	.001
PLT (10^9^/L)	112.00 (68.75–153.00)	219.00 (189.00–292.00)	<.001
AST (U/L)	413.20 (160.88–1057.00)	20.60 (18.40–29.40)	<.001
ALT (U/L)	499.75 (193.75–1182.70)	23.400 (16.30–39.70)	<.001
TB (μmol/L)	348.75 ± 138.07	11.84 ± 3.63	<.001
Crea (μmol/L)	68.70 (60.73–84.23)	64.300 (55.70–81.00)	.474
INR	2.25 ± 0.87	N/A	N/A

ACLF = acute-on-chronic liver failure, ALT = alanine transaminase, AST = aspartate transaminase, Crea = creatinine, INR = international normalized ratio, HBV = hepatitis B virus, HGB = hemoglobin, NC = normal control group, NEU = neutrophil, PLT = platelet, TB = total bilirubin, WBC = white blood cell.

* Fisher test.

HBV-ACLF patients were followed up for 90 days, and HBV-ACLF patients were divided into survival (n = 30) and non-survival (n = 20) groups according to 90-day clinical outcomes. Compared with the survival group, there were no statistically significant differences in white blood cell, neutrophil, hemoglobin, alanine transaminase, aspartate transaminase, TB, creatinine, blood urea nitrogen, serum sodium, and age in the non-survival group. The platelet count (*P* = .014) and albumin levels (*P* = .037) were significantly lower in the non-survival group than in the survival group. The survival group, international normalized ratio (*P* < .001), MELD (*P* < .001), MELD-Na (*P* = .013), and Chinese Group on the Study of Severe Hepatitis B-II score (*P* < .001) were significantly higher in the non-survival group than in the survival group (Table 2).

**Table 2:** Baseline characteristics of patients with HBV-ACLF between survival group and non-survival group.

Features/groups	Survival (n = 30)	Non-survival (n = 20)	*P*-value
Sex (male/female)	24/6	15/5	.736*
Age (years)	50.23 ± 12.52	51.60 ± 12.61	.708
WBC (10^9^/L)	6.88 (5.94–8.79)	6.61 (4.64–9.71)	.400
NEU (10^9^/L)	4.57 (3.84–6.52)	4.61 (2.99–7.33)	.649
HGB (g/L)	132.07 ± 19.34	122.65 ± 24.29	.135
PLT (10^9^/L)	137.60 ± 70.34	92.90 ± 43.11	.014
AST (U/L)	349.20 (160.88–1103.30)	653.75 (149.65–898.90)	.968
ALT (U/L)	458.20 (197.50–1182.70)	654.30 (149.65–898.90)	.937
Alb (g/L)	32.77 ± 5.42	30.12 ± 3.28	.037
TB (μmol/L)	319.67 ± 125.21	392.36 ± 147.98	.068
Crea (μmol/L)	69.05 (62.10–91.15)	67.25 (56.63–80.48)	.411
BUN	4.76 (3.41–6.37)	4.29 (3.25–7.63)	.953
Na (mmol/L)	137.45 (134.63–139.95)	136.25 (133.45–138.98)	.251
INR	1.75 (1.550–2.11)	2.67 (2.28–3.12)	<.001
MELD	22.21 ± 3.18	27.12 ± 5.50	<.001
MELD-Na	16.40 (13.73–24.89)	25.19 (20.41–29.17)	.013
COSSH-II	6.92 ± 0.59	7.73 ± 0.87	<.001

ACLF = acute-on-chronic liver failure, Alb = albumin, ALT = alanine transaminase, AST = aspartate transaminase, BUN = blood urea nitrogen, COSSH = Chinese Group on the Study of Severe Hepatitis B, Crea = creatinine, HBV = hepatitis B virus, HGB = hemoglobin, INR = international normalized ratio, MELD = model for end-stage liver disease, MELD-Na = model for end-stage liver disease derivations incorporating serum sodium, Na = serum sodium, NEU = neutrophil, PLT = platelet, TB = total bilirubin, WBC = white blood cell.

* Fisher test.

### 3.2. Quantification protein detection

**Figure 1.**
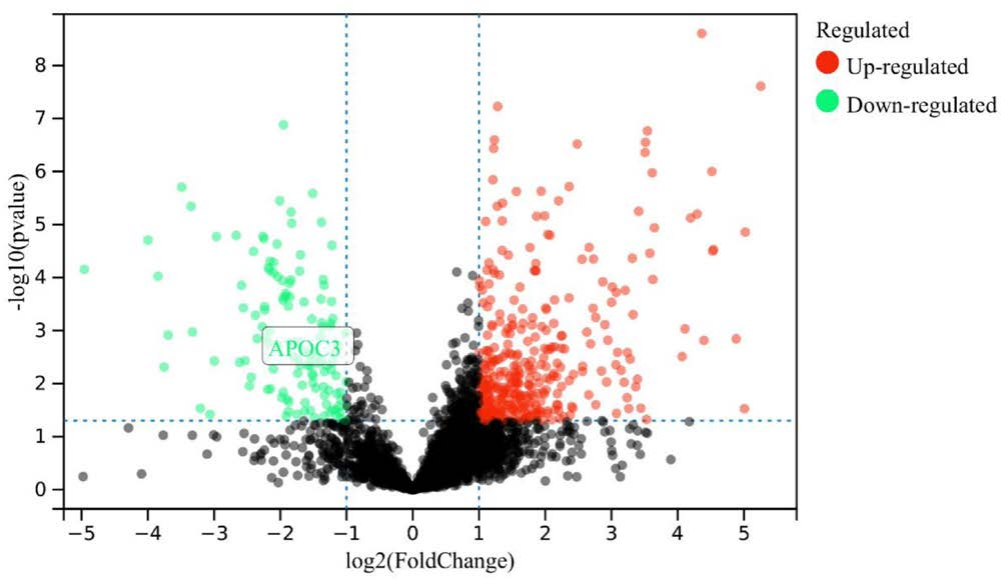
Volcano plot of differentially expressed proteins in HBV-ACLF group compared to normal control group. The red dot indicates upregulated proteins, the green dot indicates downregulated proteins, and gray dot indicates unchanged proteins. ACLF = acute-on-chronic liver failure, HBV = hepatitis B virus.

A spectral library of plasma proteins was obtained from ten HBV-ACLF patients and 5 healthy volunteers using DIA-based quantitative proteomics. The screening criteria for significantly DEPs were fold-change (|FC|) ≥ 2 and *P* < .05. The MSstats software package was used to analyze the DEPs. Through statistical analysis, 247 DEPs were identified in the serum of patients in the HBV-ACLF and control groups. A total of 148 proteins were upregulated and 99 proteins were downregulated in the HBV-ACLF group compared with those in the normal group, as reported in our previous studies.^[21]^ The purpose of this study was to explore APOC3 expression (Fig. 1).

### 3.3. GO enrichment analysis

**Figure 2.**
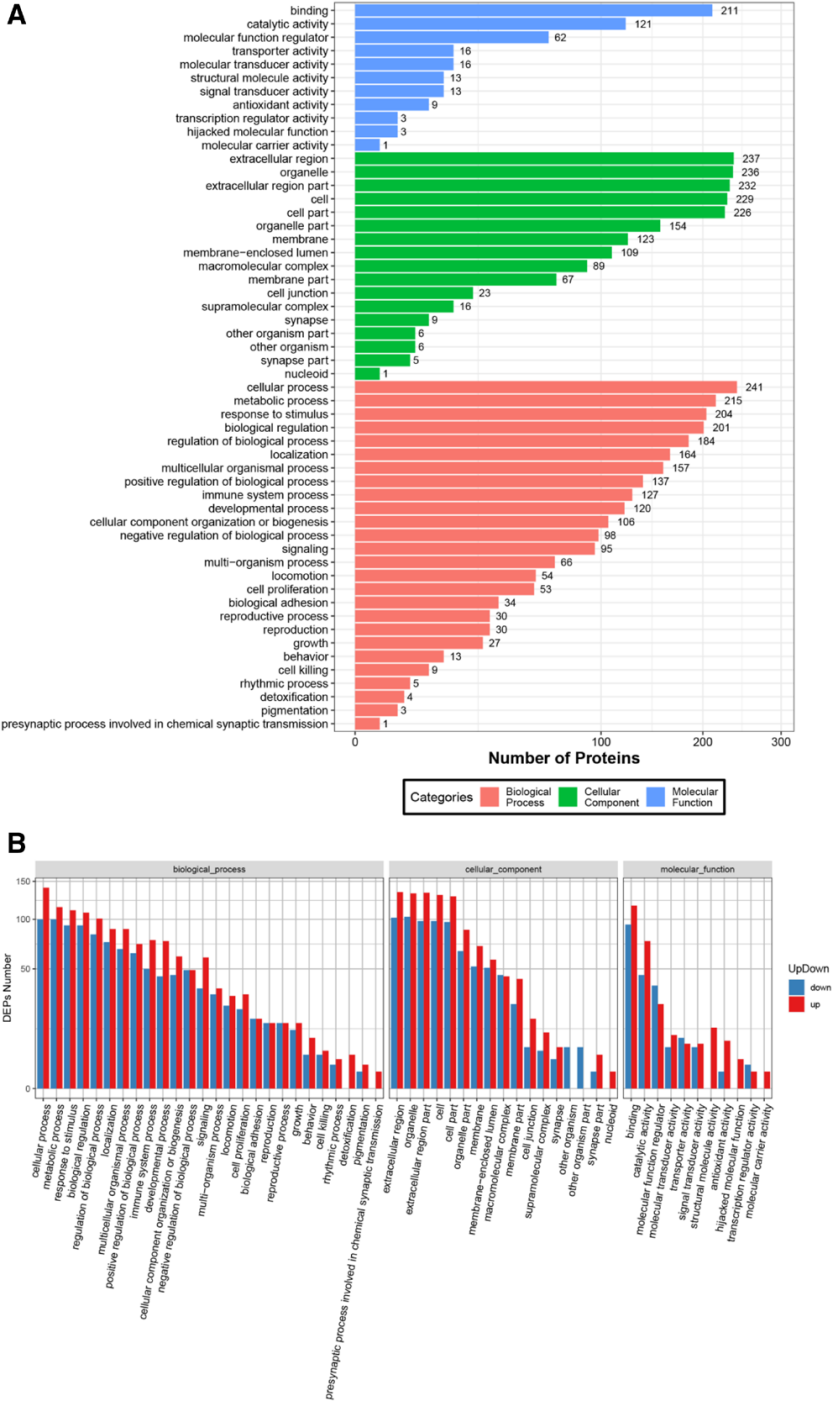
(A) GO functional classification on differentially expressed proteins in HBV-ACLF group compared to normal control group. The x-axis is the number of the differentially expressed proteins. The y-axis is GO terms. Orange indicates biological processes, green indicates cellular component, and blue indicates molecular function. (B) GO functional classification map of the differentially expressed proteins in HBV-ACLF group compared to normal control group. The x-axis is GO terms. The y-axis is the number of the differentially expressed proteins. Red indicates upregulated proteins. Blue indicates down-regulated proteins. ACLF = acute-on-chronic liver failure, GO = Gene Ontology, HBV = hepatitis B virus.

The Blast2GO software was used for enrichment analysis to evaluate the functional significance of the 247 DEPs between HBV-ACLF and normal controls. We generated GO functional classification maps to represent all DEPs and to discriminate between upregulated and downregulated proteins (Fig. 2). The results indicated that upregulated and downregulated proteins were mainly involved in biological processes, including cellular processes, metabolic processes, responses to stimuli, and biological regulation. The results indicated that the DEPs were mainly involved in cellular components, including the extracellular region, organelles, extracellular region, cells, and cell parts. The results indicated that these DEPs are mainly involved in molecular functions, including binding, catalytic activity, and regulation.

### 3.4. PPI network

**Figure 3.**
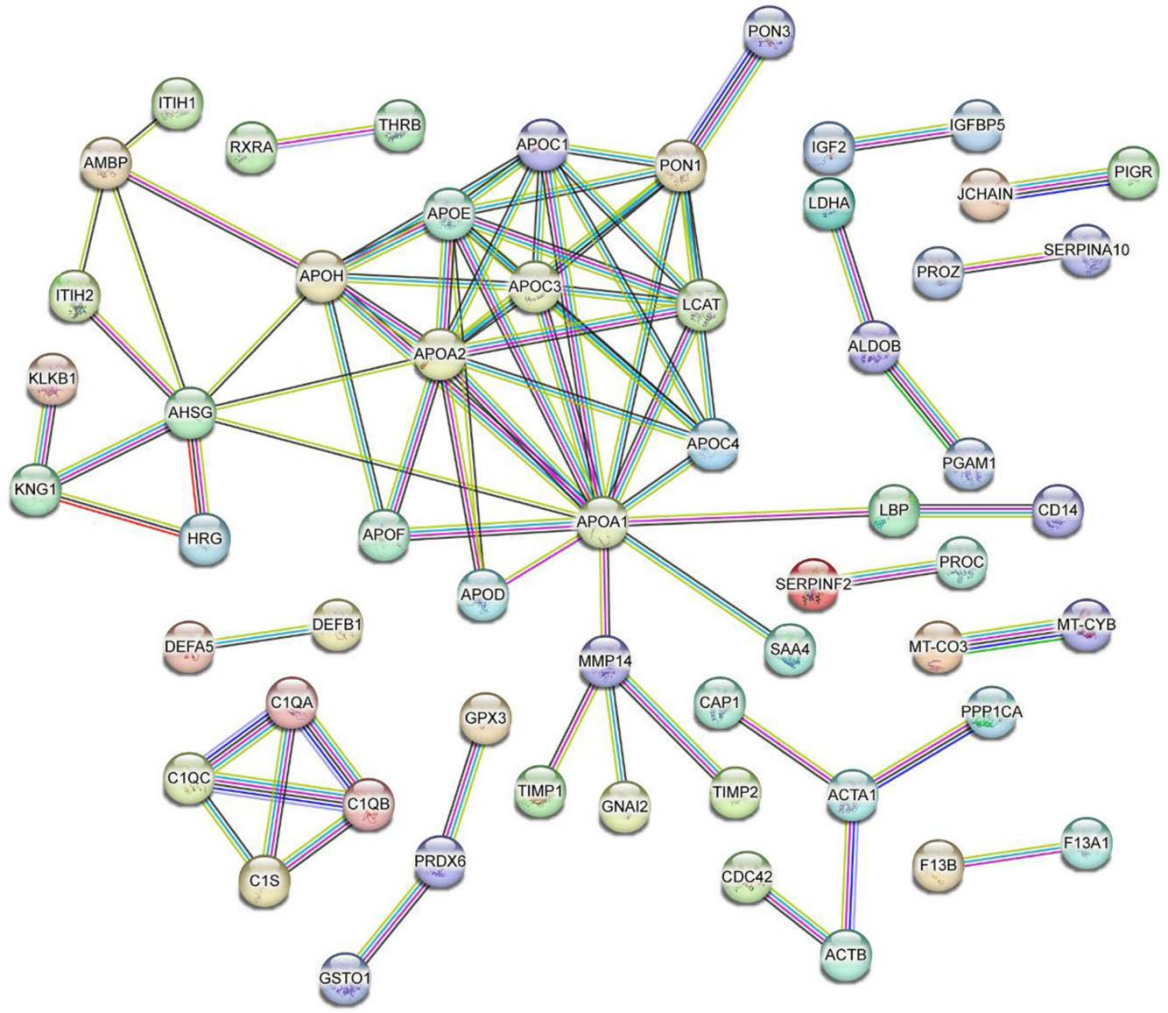
Differential expressed proteins interaction network diagram.

The DEPs were imported into the STRING online database (STRING 11.0). The PPI network showed that α1-microglobulin precursor (AMBP), apolipoprotein A1 (APOA1), apolipoprotein A2, APOC3, α2-HS glycoprotein, apolipoprotein H, paraoxonase-1, and lecithin cholesterol acyl transferase were located at the core of the network (Fig. 3). Cytoscape software analysis showed that AMBP, APOC3, and α2-HS glycoprotein had higher node degrees, particularly APOC3, indicating that APOC3 had more biological functions. The DIA results showed that the expression level of APOC3 was lower in patients with HBV-ACLF than in normal controls. To validate this hypothesis, APOC3 was screened in further studies.

### 3.5. ELISA verification

**Figure 4.**
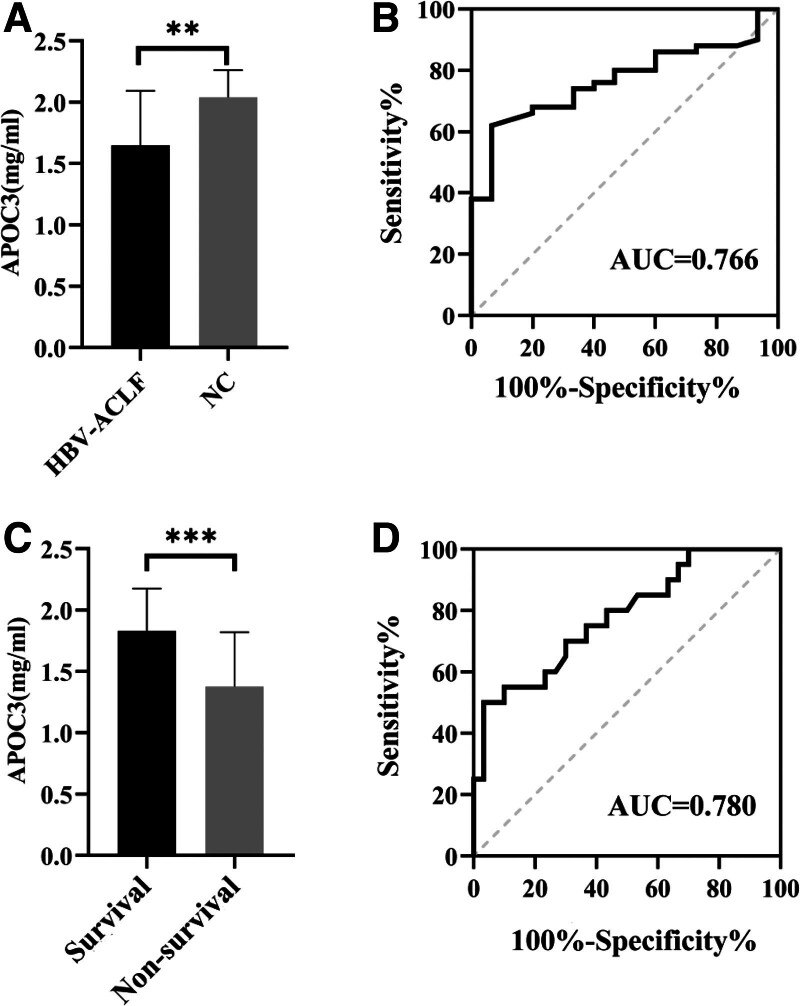
(A) The expression of APOC3 between HBV-ACLF and normal controls. ** *P* < .001. (B) ROC of APOC3 in normal group and HBV-ACLF group, AUC = 0.766. (C) The expression of APOC3 between survival group and non-survival group. ***: *P* < .0001. (D) ROC of APOC3 in survival group and non-survival group, AUC = 0.780. ACLF = acute-on-chronic liver failure, APOC3 = apolipoprotein C3, AUC = area under the curve, HBV = hepatitis B virus.

The ELISA results showed that the plasma concentration of APOC3 in patients with HBV-ACLF was 1.65 ± 0.44 mg/mL, which was obviously lower than the normal controls (2.04 ± 0.22 mg/mL) (*P* < .001, Fig. 4A). According to the ELISA results, the area under the ROC curve between the HBV-ACLF and control groups was 0.766 (Fig. 4B), with a sensitivity of 62.0% and specificity of 93.3%. We further explored whether APOC3 was associated with short-term prognosis in patients with HBV-ACLF. Patients with HBV-ACLF were divided into survival (n = 30) and non-survival (n = 20) groups according to 90-day clinical outcomes. The plasma concentration of APOC3 in the non-survival group was 1.38 ± 0.44 mg/mL, which was obviously lower than the survival group (1.83 ± 0.35 mg/mL) (*P* < .001, Fig. 4C). The area under the ROC curve between the survival and non-survival groups was 0.780 (Fig. 4D) with a sensitivity of 50.0% and specificity of 96.7%.

## 4. Discussion

The definition of ACLF varies between East and West, but it is a complex syndrome characterized by acute decompensation of liver function, rapid disease progression, organ failure(s), and a high short-term mortality rate (28-day mortality of 32%).^[22]^ Chronic HBV infection is the leading cause of ACLF in China.^[1,3]^ Immune dysfunction is central to HBV-ACLF pathogenesis and outcome, with an initial excessive systemic inflammatory response that can contribute to organ failure(s) and mortality.^[23]^ Metabolomic and proteomic studies have revealed that some proteins are associated with chronic hepatitis B infection and HBV-ACLF progression.^[21,24–26]^ In our previous study, we showed that some proteins are associated with chronic viral hepatitis B using DIA-based quantitative proteomics and bioinformatics methods.^[27]^ However, proteomic methods for the discovery of HBV-ACLF biomarkers in the serum are limited. The pathogenic mechanism of HBV-ACLF is complex and has not been fully elucidated; therefore, further research is needed. In our previous study,^[21]^ we identified 247 DEPs in HBV-ACLF patients using DIA-based quantitative proteomics compared with the normal group. Among these candidate proteins, 148 were upregulated and 99 were downregulated. In combination with bioinformatics analysis, we found that AMBP, APOA1, apolipoprotein A2, APOC3, apolipoprotein H, paraoxonase-1, and lecithin cholesterol acyl transferase are mainly involved in lipoprotein metabolism. These DEPs may play important roles in the pathogenesis of HBV-ACLF and require further investigation.

Systemic inflammatory responses play a key role in the pathophysiology of HBV-ACLF. The pathophysiology of HBV-ACLF is associated with persistent inflammation, immune dysregulation with initial generalized immune activation, systemic inflammatory response syndrome, and subsequent sepsis due to immune paralysis.^[28,29]^ A dysbalanced immune function plays an important role in the development of HBV-ACLF. Accumulating evidence suggests that apolipoproteins play important roles in cell differentiation, tissue repair, and immune regulation.^[30,31]^ Previous studies have shown a strong link between abnormal lipid metabolism and inflammation.^[32]^ The liver is an important site for apolipoprotein synthesis and degradation and is involved in lipid metabolism. Lipid metabolism is mainly processed in the liver, and lipids play a key role in the physiological processes in the liver and in the pathological progression of many diseases. Deficiency of APOA and APOB is a manifestation of advanced chronic liver disease, and advanced chronic liver disease itself can lead to decreased synthesis of APOA and APOB, resulting in decreased expression levels.^[33]^ In this study, we found that the expression level of APOC3 in the HBV-ACLF group was lower than that in normal controls, and our results were consistent with those of previous studies.^[24]^ Further research revealed that the expression of APOC3 in the non-survival group was lower than that in the survival group. The result indicates that APOC3 prognostic ability for patients with HBV-ACLF, and it indicated that APOC3 can be used as a prognostic biomarker in patients with HBV-ACLF. Studies have shown that HBV is involved in the synthesis and metabolism of apolipoproteins, including APOA1, APOB and APOC3^.[34–36]^ The molecular mechanism of APOC3 in the pathogenesis of HBV-ACLF is unknown, and its role has not yet been described and requires further intensive study.

APOC3 is a 79-amino-acid glycoprotein and a very low-density lipoprotein component that plays an important role in lipoprotein metabolism and is involved in sterile inflammatory response and organ damage.^[37,38]^ APOC3 is primarily expressed in the liver and intestine, especially in the liver, and plays a key role in regulating triglyceride-rich lipoprotein metabolism. Relevant studies have shown that APOC3 is not only involved in the occurrence and development of various diseases, such as cardiovascular diseases and glucose and lipid metabolism disorders, but also participates in other physiological and pathophysiological processes. High expression of APOC3 is an independent risk factor for some diseases such as cardiovascular disease, atherosclerosis, and nonalcoholic fatty liver disease. Previous studies have found that transgenic mice with hepatic overexpression of APOC3 are more likely to develop nonalcoholic steatohepatitis and hepatic insulin resistance.^[39]^ Recent studies have shown that APOC3 is involved in regulating inflammatory responses and tissue damage, and silencing APOC3 can alleviate lipopolysaccharide-induced acute lung injury by inhibiting toll-like receptor signaling pathways.^[40]^ The liver is an important site of lipid protein metabolism and APOC3 synthesis. When ACLF occurs, a large amount of liver tissue necrosis inevitably leads to abnormal lipid metabolism including lipid protein synthesis. In this study, we found that the plasma APOC3 concentrations in patients with HBV-ACLF were significantly lower than those in the healthy control group, especially in the death group. Therefore, up to a certain point, the plasma concentration of APOC3 can reflect the severity of HBV-ALCF and guide clinical treatment. However, further research is required to confirm.

This study had some limitations. This was a single-center study, with a relatively small sample size. A multicenter study with a large sample size is needed to validate our results in future studies.

In summary, this study suggests that APOC3 is associated with short-term prognosis of patients with HBV-ACLF and can be used as a prognostic biomarker in patients with HBV-ACLF. The pathophysiological mechanism of APOC3 involvement in HBV-ACLF regulation remains unclear and further research is needed.

## Author contributions

**Conceptualization:** Wen Chen.

**Data curation:** Bo Wang, Li Qiang, Geng Zhang.

**Formal analysis:** Bo Wang, Li Qiang.

**Investigation:** Gang Wu.

**Methodology:** Gang Wu, Cunliang Deng.

**Software:** Yunjian Sheng.

**Supervision:** Shan Zeng, Qian Zhang.

**Writing – original draft:** Bo Wang.

**Writing – review & editing:** Shan Zeng, Qian Zhang.
